# The synergistic mechanism of fibroblast growth factor 18 and integrin β1 in rat abdominal aortic aneurysm repair

**DOI:** 10.1186/s12872-022-02851-y

**Published:** 2022-09-17

**Authors:** Yilong Guo, Ren Wei, Yuan He, Hongpeng Zhang, Jianqing Deng, Wei Guo

**Affiliations:** 1 Medical School of Chinese PLA, Beijing, China; 2 Department of Vascular and Endovascular Surgery, The First Medical Centre of Chinese PLA General Hospital, 28#, Fuxing Road, Beijing, 100853 China

**Keywords:** Abdominal aortic aneurysm, Fibroblast growth factor 18, Integrin β1, Biological repair, Smooth muscle cells, Endothelial cells

## Abstract

**Background:**

Abdominal aortic aneurysms have a high mortality rate. While surgery is the preferred treatment method, the biological repair of abdominal aortic aneurysms is being increasingly studied. We performed cellular and animal experiments to investigate the simultaneous function and mechanism of fibroblast growth factor 18 and integrin β1 in the biological repair of abdominal aortic aneurysms.

**Methods:**

Endothelial and smooth muscle cells of rat arteries were used for the cellular experiments. Intracellular integrin β1 expression was regulated through lentiviral transfection. Interventions with fibroblast growth factor 18 were determined according to the experimental protocol. Several methods were used to detect the expression of elastic fiber component proteins, cell proliferation, and migratory activity of endothelial and smooth muscle cells after different treatments. For animal experiments, abdominal aortic aneurysms were induced in rats by wrapping the abdominal aortae in sterile cotton balls soaked with CaCl_2_ solution. Fibroblast growth factor 18 was administered through tail vein injections. The local expression of integrin β1 was regulated through lentiviral injections into the adventitia of the abdominal aortic aneurysms. The abdominal aortae were harvested for pathological examinations and tensile mechanical tests.

**Results:**

The expression of integrin β1 in endothelial and smooth muscle cells could be regulated effectively through lentiviral transfection. Animal and cellular experiments showed that fibroblast growth factor 18 + integrin β1 could improve the expression of elastic fiber component proteins and enhance the migratory and proliferative activities of smooth muscle and endothelial cells. Moreover, animal experiments showed that fibroblast growth factor 18 + integrin β1 could enhance the aortic integrity to withstand stretch of aortic aneurysm tissue.

**Conclusion:**

Fibroblast growth factor 18 + integrin β1 improved the biological repair of abdominal aortic aneurysms in rats by increasing the expression of elastic proteins, improving the migratory and proliferative abilities of endothelial and smooth muscle cells, and improving aortic remodeling.

**Supplementary Information:**

The online version contains supplementary material available at 10.1186/s12872-022-02851-y.

## Background

“Aortic aneurysm” refers to the pathological expansion of the aortic wall. When the diameter of an expanded aorta exceeds 50% of the normal aorta, it is diagnosed as an aortic aneurysm [1]. In the Western population, the morbidity of abdominal aortic aneurysms (AAAs) is approximately 7.1 to 25.8 per 100,000 people per year [2]. Approximately 90% of patients with AAAs have no obvious symptoms before an aneurysm rupture occurs. Moreover, the mortality rate is above 80% after aneurysm rupture [3]. Therefore, early detection and treatment are crucial for treating AAAs.

In order to improve the diagnosis and treatment of AAAs, their pathogenesis should be elucidated. The main pathophysiological mechanisms of aortic aneurysm formation are the apoptosis of smooth muscle cells (SMCs) and endothelial cells (ECs) and extracellular matrix degradation, resulting in the thinning and dilation of the aortic wall [4]. Elastic fibers are the major components of the aortic wall that maintain its integrity [5]. Fibroblast growth factor 18 (FGF18) is a member of the fibroblast growth factor family and is mainly expressed in the lungs, pancreas, gastrointestinal tract, and vascular wall [6]. Studies have shown that FGF18 promotes the expression of elastic fiber component proteins (including elastin [ELN], microfibril-1, and fibulin-5) in the aortic wall [7–11]. Although FGF18 can promote the expression of elastic fiber component proteins in AAA walls [12], its effect on the repair of AAAs is insignificant [13]. This is possibly because elastic fiber component proteins need to form elastic fibers to perform biological repair, and these elastic fibers need to be firmly attached to the cellular surface via proteins that are in contact with the cytoskeleton to allow the cells to sense mechanical cues. Improving mechanosensing is thought to improve cellular function and survival, combating aneurysm formation [14]. This implies that improving the connection between elastic fibers and the cytoskeleton could facilitate the repair of AAAs.

Integrin β1 (Itgβ1) is a transmembrane protein that plays a key role in the connection between the cytoskeleton and extracellular matrix [15, 16]. A study by Cheuk et al. showed that the expression of Itgβ1 is considerably reduced in aortic aneurysm tissue [17]. Itgβ1-knockout mice have shown remarkable aortic dilatation, which suggests that this receptor plays a key role in maintaining aortic wall stability [18, 19]. Therefore, this study innovatively applied FGF18 + Itgβ1 to repair AAAs and explored the feasibility and mechanisms of this therapeutic regimen.

## Methods

### Cell experiments

Rat cerebral arterial ECs and aortic SMCs were selected for the cellular experiments, both of which were purchased from Procell (Wuhan, China). The cells were grouped as follows: normal cell group (NC); Itgβ1 knockdown control group (LV-Con-RNAi); Itgβ1 knockdown group (LV-Itgβ1-RNAi); Itgβ1 overexpression control group (LV-Con); and Itgβ1 overexpression group (LV-Itgβ1).

#### Cell culture and lentiviral transfection

Arterial ECs and SMCs were cultured in 1% penicillin/streptomycin, sodium pyruvate, glutamine, 10% fetal bovine serum (FBS), and 90% high-glucose medium at 37 ℃ in 5% CO_2_. The plasmids for the overexpression or knockdown of Itgβ1 were constructed and packaged with lentivirus. The lentivirus vectors were subsequently used to transfect the arterial ECs and SMCs. The efficacy of the infections was determined 48 h after transfection.

#### FGF18 treatment

Exogenous FGF18 treatment was performed according to different groups. In the ECs, FGF18 solution of different concentrations (0, 25, 50, 100, and 200 ng/mL) was used to regulate the expression of ELN, fibrillin-1 (FBN1), and fibulin-5 (FBLN5). The expression of these proteins in different groups was determined at different time points (0, 6, 12, 24, and 48 h). Subsequently, FGF18 solution (100 ng/mL) was used to treat the ECs in the NC, NC + LV-Con, NC + LV-Itgβ1-RNAi, and NC + LV-Itgβ1 groups. The expression levels of Itgβ1, ELN, FBN1, and FBLN5 in these groups were detected using western blot (WB) assays 24 h after FGF18 treatment. In the SMCs, FGF18 solution of varying concentrations (0, 100, 200, and 400 ng/mL) was used to treat the cells separately. The expression of ELN in different groups was determined at different time points (0, 12, 24, and 48 h). Subsequently, an FGF18 solution of different concentrations (100 and 200 ng/mL) was used to treat the SMCs in the NC, NC + LV-Con, NC + LV-Itgβ1-RNAi, and NC + LV-Itgβ1 groups separately. The expression levels of Itgβ1, ELN, fibrillin-1, and FBLN5 in different groups were detected using WB assays 24 h after FGF18 treatment.

#### Western blot assay

The cells were lysed with a RIPA lysis buffer (Beyotime, Shanghai, China) containing a 1% protease inhibitor. The protein extraction was quantitatively analyzed using the BCA protein analysis kit (Beyotime, Shanghai, China). The protein sample was added to a 10% sodium dodecyl sulfate–polyacrylamide gel electrophoresis (SDS-PAGE) gel for electrophoretic separation and transferred to a polyvinylidene difluoride (PVDF) membrane. After being blocked, the PVDF membranes were incubated with monoclonal primary antibodies at 4 °C overnight. Next, the membrane was incubated with secondary antibodies for 2 h. The WB bands were visualized using Pierce™ ECL (Thermo Fisher Scientific Inc., Shanghai, China) and analyzed using ImageJ software (https://imagej.en.softonic.com/mac).

#### RNA extraction and RT-qPCR

TRIzol™ Reagent (Invitrogen, Shanghai, China) was used to extract RNA. The RNA samples were then reverse transcribed into cDNA using a reverse transcription kit (TOYOBO, Shanghai, China). Real-time quantitative reverse transcription-polymerase chain reaction (RT-qPCR) was performed on the Applied Biosystems™ 7500 real-time PCR system using the SYBR® Green Realtime PCR Master Mix (TOYOBO, Shanghai, China). All the primers were designed and synthesized by TOYOBO (Shanghai, China). The specific primer sequences used in this study are presented in Table 1.

**Table 1:** Primer sequences used in this study

Gene	Forward	Reverse
Itgβ1	5′-GACCTGCCTTGGTGTCTGTGC-3′	5′-AGCAACCACACCAGCTACAAT-3′
Elastin	5′-GCCCTGGGATATCAAGGTGG-3′	5′-CACTGGCCTGTTGTCCCC-3′
Fibulin-5	5′-CTCTGCAGTGGCTCCCAGC-3′	5′-CCACAGTGCCAGGATGGTGA-3′
Fibrillin-1	5′-TCAGATGGCCGCTACTGCAA-3′	5′-CAGTGTTCACGCATCGTCCG-3′

#### Cell Counting Kit-8 assay

Cell proliferation was detected using the Cell Counting Kit (CCK)-8 assay (Dojindo, Kumamoto, Japan). ECs and SMCs were inoculated into 96-well plates (ECs, 5 × 103 cells/well; SMCs, 2 × 103 cells/well). The cells were cultured at 37 °C in 5% CO_2_. After the cells had adhered to the wall, FGF18 (100 ng/mL) was added to the experimental group, and cell culture was continued for 6–48 h. CCK-8 reagent (10 µL/well) was added to each well, and absorbance was measured at 450 nm after 2 h of incubation. The absorbance results were subsequently used to determine cell proliferation.

#### Transwell migration assay

Cell migration was detected using the Transwell migration assay. A Transwell insert chamber with an 8-μm pore size membrane (Corning, Shanghai, China) was used for the assay. EC (4 × 104 cells/mL) and SMC (5 × 104 cells/mL) solutions and FGF18 (100 ng/mL) solution were transferred into the upper chamber separately. As a chemoattractant, 500 mL of medium containing 20% FBS were added into the lower chamber. After 6–24 h of culture, the medium in the Transwell chamber was discarded. The cell culture was then washed with phosphate-buffered saline (PBS), and non-migrating cells in the upper chamber were gently wiped off. The chamber was placed in 4% paraformaldehyde for 15 min and 0.1% crystal violet dye for 30 min. Five visual fields of the chamber were randomly observed, and the cells were counted.

### Animal experiments

Sixty adult Sprague–Dawley (SD) rats (SBF Biotechnology Co., Ltd, Beijing, China) (males weighing 350–400 g) were randomly divided into five different equally sized groups. They were grouped into the Sham, AAA, AAA + FGF18, AAA + LV-Itgβ1, and AAA + LV-Itgβ1 + FGF18 groups.

#### Rat AAA model

Previous studies showed that CaCl_2_ solution could disrupt the elastic network within the aortic media by activating the inflammatory response through calcium precipitation, ultimately leading to AAA [20]. So, AAA were induced in rats by wrapping the abdominal aortae in sterile cotton balls soaked with CaCl_2_ solution. Anesthesia was administered via intraperitoneal injections of 3% pentobarbital sodium. A middle incision was made in the abdomens to expose the abdominal aortae between the renal and bilateral iliac arteries. The abdominal aortae were isolated from surrounding tissues with sterile rubber strips and wrapped in sterile cotton balls soaked in CaCl_2_ solution (0.8 mol/L) [21]. The abdominal aortae of the Sham group were wrapped in sterile cotton balls soaked in PBS. The abdominal cavities were rinsed with sterile saline three times and closed layer by layer 30 min later. The construction of the AAA model is shown in Additional file 1: Fig. S1.

All the rats were fed a normal diet for 14 days after the operations. The maximum diameters of the abdominal aortae were detected using an abdominal ultrasound. It is reported that the normal size of a rat’s abdominal aorta is approximately 3–4 mm [21]. However, the abdominal aortic diameter is greatly affected by age and weight, making it an unideal diagnostic indicator of AAA. Instead, the abdominal aortic dilation rate was used as an indicator of AAA diagnosis in this study as follows: Abdominal aortic dilation rate = {(maximum diameter at measurement—maximum diameter on day 0) / maximum diameter on day 0} × 100%. A maximum abdominal aortic dilation rate ≥ 40% was the criterion set for successful AAA modeling. The rats in the AAA + LV-Itgβ1 and AAA + FGF18 + LV-Itgβ1 groups were then transfected with LV-Itgβ1. The AAAs were exposed along the abdominal incisions, and a solution containing LV-Itgβ1 was injected under the aortic adventitia on both sides of the aneurysms. The abdominal incisions were sutured layer by layer 30 min later. Rats in the AAA + FGF18 + LV-Itgβ1 and AAA + FGF18 groups received tail vein injections of FGF18 (5 μg) on days 14, 17, 20, 23, and 25 after the operations.

The maximum diameters of the abdominal aortae were measured by ultrasound on days 0, 14, and 28 after the surgeries. The rats were then sacrificed by cervical dislocations, and the abdominal aortae were harvested. If the rats died during the course of the experiment, the abdominal aortae were harvested immediately. The abdomens were opened along the previously made incisions, and the aortae between the renal and iliac arteries were harvested (ensuring that at least 5 mm of normal aortic tissue were present on both ends of the aneurysms). Five samples from each group were left for the tensile mechanical tests (to test how far the aorta can be stretched in length before breaking), and the remaining samples were fixed in 10% formaldehyde solution for 48 h. The samples were subsequently embedded in paraffin and sectioned.

#### Immunohistochemical assay

Immunohistochemical staining of the aortic tissue was performed to detect Itgβ1 expression in different groups after lentiviral transfection. Paraffin-embedded aortic tissue sections were dewaxed and hydrated, and antigen repair was performed. Anti-Itgβ1 was added, and the mixture was incubated overnight at 4 °C. A secondary antibody was added and further incubated for 60 min. The sections were then soaked in hematoxylin solution for 8 min and sealed with neutral resin, after which the tissue samples were observed.

#### Hematoxylin and eosin staining

AAA tissues were fixed with formaldehyde, embedded in paraffin, and cut into 4-μm-thick sections. The tissue sections were subsequently stained with hematoxylin and eosin (HE) solution.

#### Elastic Verhoeff-Van Gieson staining

AAA tissues were fixed with formaldehyde, embedded in paraffin, and cut into 4-μm-thick sections. They were then stained with elastic Verhoeff-Van Gieson (EVG) dye.

#### Tensile mechanical assay

A Microtester 5848 micro force material testing machine (Chinese Academy of Science, Beijing, China) was used to detect the aortic integrity to withstand stretch of different groups. A 5-N mechanical sensor with an initial gauge distance of 10 mm and a load threshold of 3 N was used. The upper and lower ends of the aortae were fixed, and the specimens were slowly pulled at 10 mm/min (to test how far the aorta can be stretched in length before breaking). Maximum tension under different displacements was recorded until the aortae were broken. A displacement-tension curve was then plotted according to the recorded data.

### Statistical analysis

SPSS (version 20.0, IBM Corp., Armonk, NY, USA) was used for data analysis. Continuous variables were expressed as mean ± standard deviation. One-way analysis of variance, *t*-test, chi-square test, or Fisher’s exact test was used to compare the differences between groups as appropriate. *p* < 0.05 was considered statistically significant.

## Results

### Cell experiments

#### Lentiviral transfection

Aortic ECs and SMCs from rats were transfected with LV-Itgβ1-RNAi and LV-Itgβ1, respectively. The transfection efficacy was determined 48 h after transfection. The results showed that successful transfection was achieved by transfecting with the two vectors. The fluorescence abundance of the NC + LV-Itgβ1 group was the highest, whereas that of the NC + LV-Itgβ1-RNAi group was the lowest. There was no significant difference between the NC + LV-Con-RNAi and NC + LV-Con groups (Additional file 1: Fig. S2).

WB and RT-qPCR assays were performed to detect Itgβ1 expression changes in ECs and SMCs after lentiviral transfection. The results showed that Itgβ1 expression was not significantly different between the NC, NC + LV-Con, and NC + LV-Con-RNAi groups (*p* > 0.05). The Itgβ1 expression of the NC + LV-Itgβ1 group was the highest, whereas that of the NC + LV-Itgβ1-RNAi group was the lowest (Additional file 1: Fig. S2).

#### The effect of FGF18 and FGF18 + Itgβ1 on the expression of elastic fiber component proteins in ECs

**Fig. 1:**
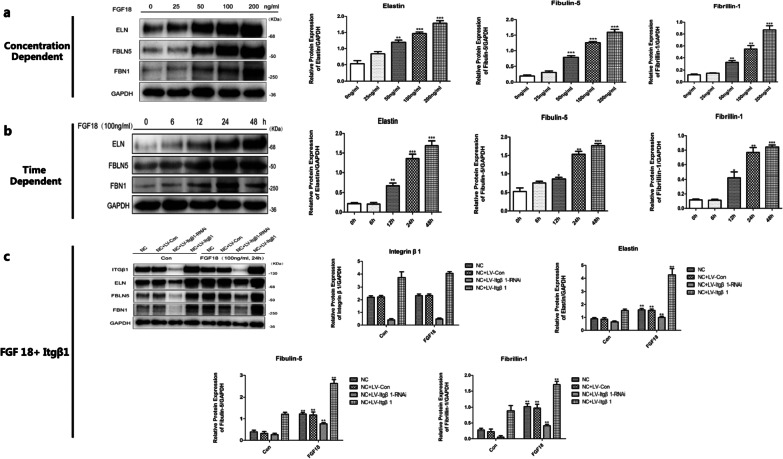
**a** FGF18 promotes the expression of elastic fiber component proteins in ECs in a concentration-dependent manner; after 24 h of treatment with FGF18 solution of different concentrations, the expression of elastin, fibulin-5, and fibrillin-1 in ECs increased with the increasing FGF18 solution concentration; **p < 0.01 versus the 0 ng/mL group. ***p < 0.005 versus the 0 ng/mL group; **b** FGF18 promotes the expression of elastic fiber component proteins in ECs in a time-dependent manner; when the concentration of the FGF18 solution was fixed at 100 ng/mL, the expression of the elastic fiber component proteins in ECs increased with the increase in treatment duration; **p < 0.01 versus the 0 h group. ***p < 0.05 versus the 0 h group; **c** Effects of FGF18 + Itgβ1 on elastic fiber component proteins and Itgβ1 expression in Ecs; the expression of elastic fiber component proteins increased in the groups treated with FGF18. After treatment with FGF18, ECs in the NC + LV-Itgβ1 group showed a higher expression of elastic fiber component proteins than the other groups; FGF18 did not affect the expression of Itgβ1; **p < 0.01 versus the control groups (groups that were not treated with FGF18)

Twenty-four hours after treatment with different concentrations of FGF18 solution, the expression of elastic fiber component proteins in the ECs increased with increasing FGF18 solution concentrations. When the concentration of the FGF18 solution was fixed at 100 ng/mL, the expression of the elastic fiber component proteins increased with the duration of the treatment. Therefore, we established that FGF18 promotes the expression of elastic fiber component proteins in ECs in a concentration- and time-dependent manner (Fig. 1). The effect of FGF18 + Itgβ1 on the expression of elastic fiber component proteins in ECs was subsequently explored. The results showed that the expression of elastic fiber component proteins increased in all four groups after FGF18 treatment. In addition, after treatment with FGF18, ECs of the NC + LV-Itgβ1 group showed a higher expression of elastic fiber component proteins than the other groups (*p* < 0.01). FGF18 did not affect the expression of Itgβ1 in any of the groups. This suggests that FGF18 and Itgβ1 act synergistically in promoting the expression of elastic fiber component proteins in ECs (Fig. 1).

#### The effect of FGF18 and FGF18 + Itgβ1 on elastic fiber component protein expression in SMCs

**Fig. 2:**
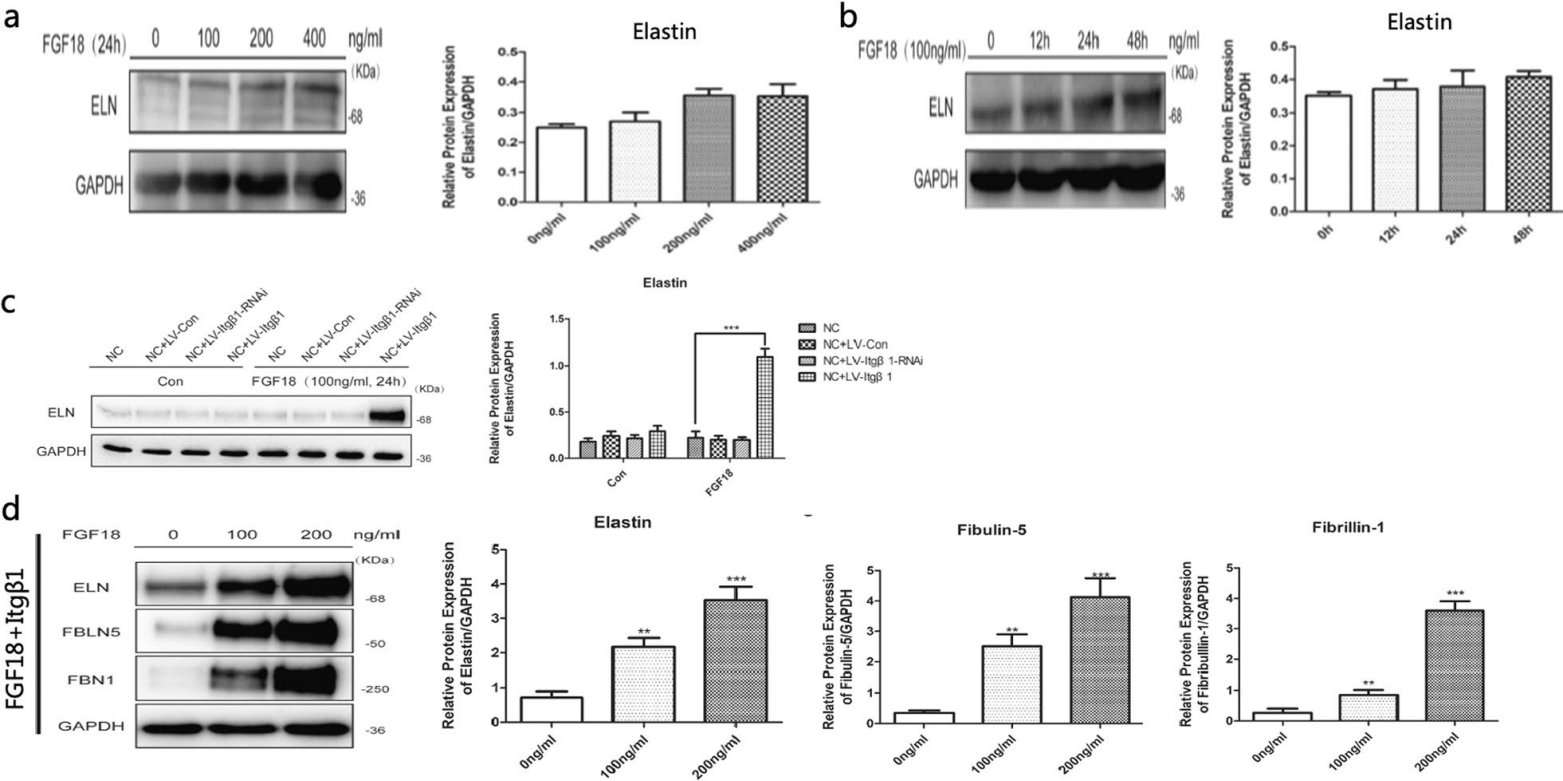
Effects of FGF18 on elastin expression in SMCs. **a** There was no significant difference in elastin expression between the SMCs treated with 100 ng/mL of FGF18 solution and the SMCs that were not treated with the FGF18 solution (*p* = 0.163); there was no significant difference in elastin expression between the SMCs treated with 200 ng/mL of FGF18 solution and the SMCs treated with 400 ng/mL of FGF18 solution (*p* = 0.379). **b** There was no significant difference in the expression of elastin at different time points after treatment with 100 ng/mL FGF18 solution (*p* > 0.05). **c** FGF18 + Itgβ1 can promote the expression of elastin; in the Con group (not treated with FGF18), there was no significant difference in elastin expression amongst the NC, NC + LV-con, NC + LV-Itgβ1-RNAi, and NC + LV-Itgβ1 groups (*p* > 0.05); there was also no significant difference in elastin expression amongst the NC, NC + LV-Con, and NC + LV-Itgβ1-RNAi groups after FGF18 treatment (*p* > 0.05); after treatment with FGF18 (100 ng/mL, 24 h), the elastin expression in SMCs in the NC + LV-Itgβ1 group was higher than that in the other three groups (*p* < 0.05). ***p < 0.001 versus the NC group (treated with FGF18); (**d**) FGF18 + Itgβ1 promoted the expression of elastic fiber component proteins in SMCs; the expression levels of elastin, fibulin-5, and fibrillin-1 in SMCs in the NC + LV-Itgβ1 group showed a positive relationship with the concentration of the FGF18 solution; The WB gel of a, b, c, and d were cut prior to hybridization with antibodies; **p < 0.05 versus the 0 ng/mL group. ***p < 0.001 versus the 0 ng/mL group

SMCs were treated with FGF18 solution of different concentrations, and the expression of ELN was detected at different time points after the treatment. The expression of ELN in the SMCs did not always increase with the FGF18 solution concentration, and this phenomenon was unaffected by the duration of treatment (Fig. 2). The effect of FGF18 + Itgβ1 on the expression of elastic fiber component proteins in SMCs was subsequently explored. The results showed that after treatment with FGF18, SMCs in the NC + LV-Itgβ1 group had a higher expression of ELN than those in the other groups (*p* < 0.05) (Fig. 2). Furthermore, the expression of ELN, FBLN5, and fibrillin-1 in the SMCs in the NC + LV-Itgβ1 group exhibited a positive relationship with the concentration of the FGF18 solution. This implies that FGF18 and Itgβ1 act synergistically to promote the expression of elastic fiber component proteins in SMCs (Fig. 2).

#### The effect of FGF18 and FGF18 + Itgβ1 on the proliferative and migratory abilities of ECs

**Fig. 3:**
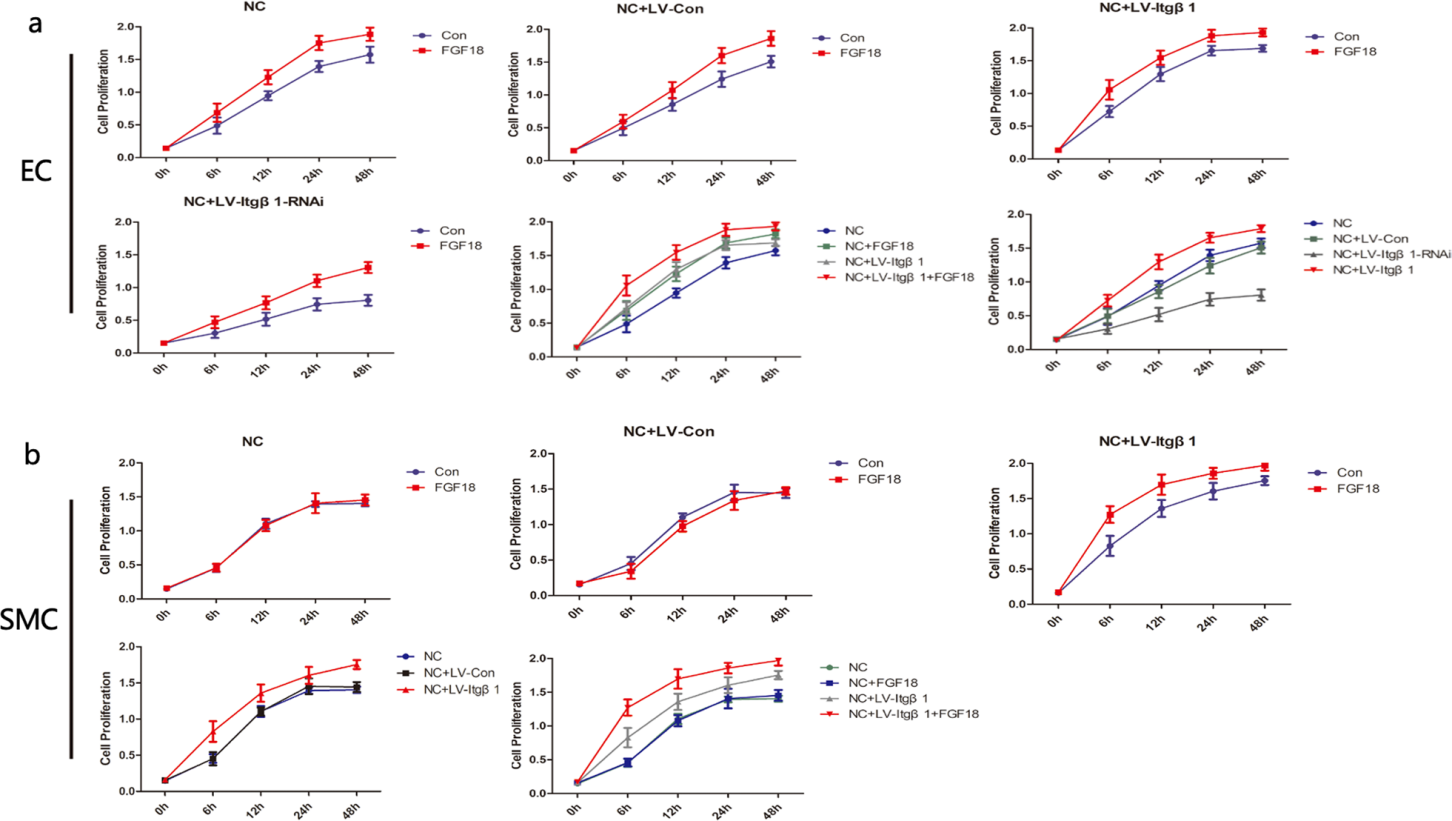
CCK-8 assay for detecting the proliferative activity of ECs and SMCs. **a** Ecs: the proliferative activity of ECs was significantly enhanced after treatment with FGF18 (*p* < 0.05); the proliferative activity of ECs was higher in the NC + LV-Itgβ1 + FGF18 group than in the NC + LV-Itgβ1, NC + FGF18, and NC groups (*p* < 0.05); amongst the groups that were not treated with FGF18, the proliferative activity of ECs in the NC + LV-Itgβ1 group was the strongest, whereas that in the NC + LV-Itgβ1-RNAi group was the weakest. **b** SMCs: FGF18 alone could not improve the proliferative activity of SMCs in the NC and NC + LV-Con groups (*p* = 0.127, 0.082); FGF18 improved the proliferative activity of SMCs in the NC + LV-Itgβ1 group (*p* = 0.032); the proliferative activity of SMCs was higher in the NC + LV-Itgβ1 group than in the NC and NC + LV-Con groups (*p* = 0.012, 0.024); the proliferative activity of SMCs was higher in the NC + LV-Itgβ1 + FGF18 group than in the NC, NC + LV-Itgβ1, and NC + FGF18 groups (*p* < 0.05)

CCK-8 and Transwell chamber migration assays were performed to explore the effects of FGF18 and FGF18 + Itgβ1 on the proliferative and migratory abilities of ECs. The results of the CCK-8 assay showed that the proliferative ability of ECs in the NC + LV-Itgβ1 + FGF18 group was considerably higher than that in the other groups (*p* < 0.05) (Fig. 3). The Transwell chamber migration assay further revealed that the migratory activity of ECs in the NC + LV-Itgβ1-RNAi group was the weakest, whereas that in the NC + LV-Itgβ1 + FGF18 group was the strongest (Additional file 1: Fig. S3). Both Itgβ1 and FGF18 could therefore improve the proliferative and migratory abilities of ECs.

#### The effect of FGF18 and FGF18 + Itgβ1 on the proliferative and migratory abilities of SMCs

CCK-8 and Transwell chamber migration assays were performed to explore the effects of FGF18 and FGF18 + Itgβ1 on the proliferative and migratory abilities of SMCs. The CCK-8 assay showed that FGF18 alone could not improve the proliferative activity of SMCs in the NC and NC + LV-Con groups (*p* = 0.127, 0.082). However, the overexpression of Itgβ1 could improve the proliferative activity of SMCs in the NC + LV-Itgβ1 group. The proliferative activity of SMCs was higher in the NC + LV-Itgβ1 + FGF18 group than in the NC + LV-Itgβ1 group (*p* = 0.013), which indicates that Itgβ1 exerts a synergistic effect with FGF18 in enhancing the proliferative activity of SMCs (Fig. 3). The Transwell chamber assay further revealed that Itgβ1 could enhance the migratory activity of SMCs. The migratory activity of SMCs was higher in the NC + LV-Itgβ1 + FGF18 group than in the other groups. This is a further indication of the synergistic effect of FGF18 and Itgβ1 in enhancing the migratory ability of SMCs (Additional file 1: Fig. S3).

### Animal experiments

#### Rat AAA models

**Fig. 4:**
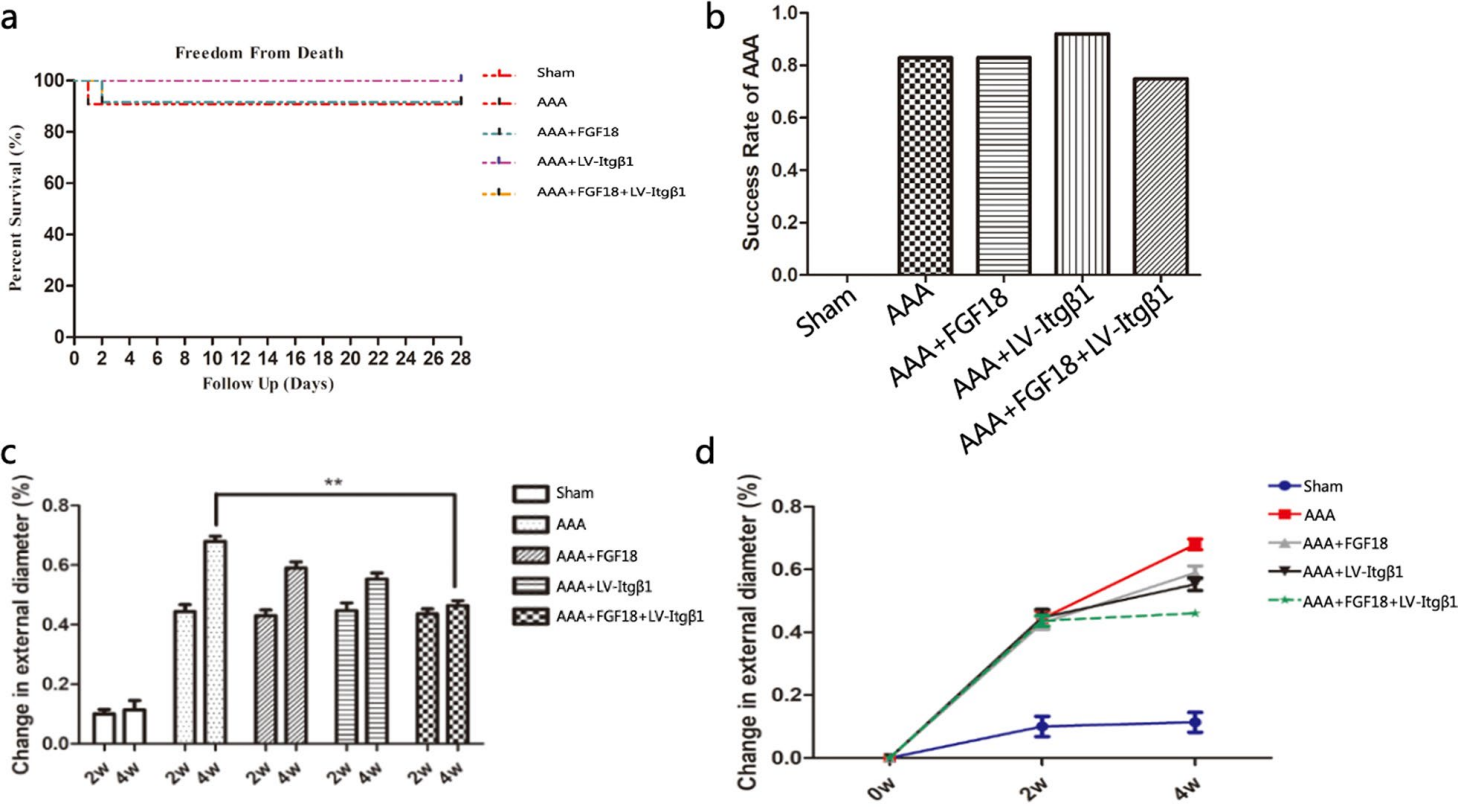
**a** Survival curves of rats in different groups; mortality of the Sham group was 0 within 4 weeks after surgery; three rats in the experimental groups died within 2 weeks after surgery, and the overall mortality of the experimental groups within 4 weeks after surgery was 6.25% (3/48). **b** The AAA incidence rate of rats in different groups: forty rats developed AAA: 10 in the AAA group, 10 in the AAA + FGF18 group, 11 in the AAA + LV-Itgβ1 group, and nine in the AAA + FGF18 + LV-Itgβ1 group; there was no significant difference in the morbidity and mortality between the experimental groups (*p* > 0.05). (**c, d**) The abdominal aorta dilation rate of different groups at 0–4 weeks after the operation. There was no significant difference in the abdominal aorta dilation rate between groups 2 weeks after the operation (*p* > 0.05); four weeks after the operation, the abdominal aorta dilation rate of the AAA + FGF18 + LV-Itgβ1 group was the lowest, whereas that of the AAA group was the highest; there was a significant difference between them (*p* < 0.01); **p < 0.01

The overall incidence rate of AAAs was 0 in the Sham group and 83.3% (40/48) in the experimental (AAA, AAA + FGF18, AAA + LV-Itgβ1, and AAA + FGF18 + LV-Itgβ1) groups. The AAA incidence rates in the animal models were 83.3% (10/12), 83.3% (10/12), 91.7% (11/12), and 75% (9/12) in the AAA, AAA + FGF18, AAA + LV-Itgβ1, and AAA + FGF18 + LV-Itgβ1 groups, respectively. The mortality rate in the Sham group 28 days after surgery was 0, whereas the overall mortality rate of the experimental groups was 6.25% (3/48) 14 days after surgery. All the deaths were caused by intestinal obstructions (one patient in each of the AAA, AAA + FGF18, and AAA + FGF18 + LV-Itgβ1 groups). The overall mortality rate between 14 and 28 days after surgery was 0 in the experimental groups. These experiments determined that abdominal aortae wrapped in sterile cotton balls soaked in a CaCl_2_ solution could safely and effectively develop AAA rat models (Fig. 4).

#### Expansion of the abdominal aorta in different groups

Two weeks after the operation, there was no significant difference in the dilation of the abdominal aortae between the experimental groups (*p* > 0.05). In contrast, the dilation rate of the abdominal aortae in the AAA + FGF18 + LV-Itgβ1 group was the lowest, whereas that of the AAA group was the highest four weeks after surgery. There was a significant difference between these two groups (*p* < 0.01). The abdominal aorta dilation rates of different groups 0–4 weeks after the operation are shown in Fig. 4.

#### Immunohistochemical staining

**Fig. 5:**
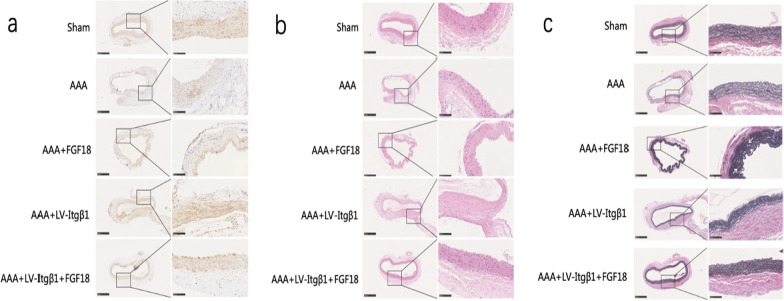
**a** Immunohistochemical staining: Itgβ1 was mainly expressed in the intima and media of the aortic wall; Itgβ1 was strongly expressed (+++) in the Sham group; Itgβ1 expression in the AAA + FGF18 and AAA groups was similar; both were weakly positive (+); compared with the AAA group, Itgβ1 expression was significantly higher in the AAA + LV-Itgβ1 and AAA + FGF18 + LV-Itgβ1 groups. Both groups were moderately positive (++). **b** HE staining: the aortic wall of the Sham group was normal; the structure of the aortic wall in the AAA group was destroyed, and rupturing of the elastic fibers and sparse arrangement were observed; the elastic fibers of the AAA + FGF18 group were not damaged to some extent; the aortic media in the AAA + LV-Itgβ1 + FGF18 group was thinner than that in the Sham group, and the overall structure of the aortic wall was relatively intact. **c** EVG staining: The structure of the abdominal aortic wall in the Sham group was normal, and the elastic fibers were continuous and regularly corrugated; the elastic fibers of the AAA group were broken and irregularly arranged; AAA + FGF18 group: the elastic fibers increased, and a sparse arrangement and local fractures were osberved; the white tip represents the irregularly arranged elastic fibers; the elastic fibers of the AAA + LV-Itgβ1 group were dense and regularly arranged, but local fractures were observed; the elastic fibers of the AAA + FGF18 + LV-Itgβ1 group were continuous and densely arranged; bar = 500 μm, 100 μm

Immunohistochemical staining was used to detect the expression of Itgβ1 in the aortic tissues of different groups (Fig. 5). Itgβ1 was mainly expressed in the intima and media of the aortic walls and was positively expressed in aortic ECs and SMCs. Itgβ1 expression was low in the AAA group and considerably higher in the AAA + LV-Itgβ1 and AAA + LV-Itgβ1 + FGF18 groups.

#### Hematoxylin and eosin staining

In the Sham group, there were no obvious structural abnormalities in the abdominal aortic walls. However, in the AAA group, the structure of the aortic wall was destroyed, and the elastic fibers were ruptured and in sparse arrangement. Compared to the AAA group, the elastic fibers in the AAA + FGF18 group were not damaged to some extent, but a disorganized aortic wall structure was still observed. The overall structure of the aortic wall in the AAA + LV-Itgβ1 + FGF18 group was relatively intact, and the elastic fibers were continuous, indicating that FGF18 + Itgβ1 can effectively promote the biological repair of aneurysm walls (Fig. 5).

#### Elastic Verhoeff-Van Gieson staining

Elastic fibers of abdominal aortae were detected through EVG staining (Fig. 5). In the Sham group, the structure of the abdominal aortic wall was normal, and the elastic fibers were continuous and regularly corrugated. In the AAA group, the elastic fibers were broken and irregularly arranged. In the AAA + FGF18 group, however, the elastic fibers increased, and a sparse arrangement and local fracture were observed. In the AAA + LV-Itgβ1 group, the elastic fibers were dense, but there were also local fractures. In the AAA + FGF18 + LV-Itgβ1 group, the elastic fibers were continuous and densely arranged.

#### Tensile mechanical test of rat AAA tissue

**Fig. 6:**
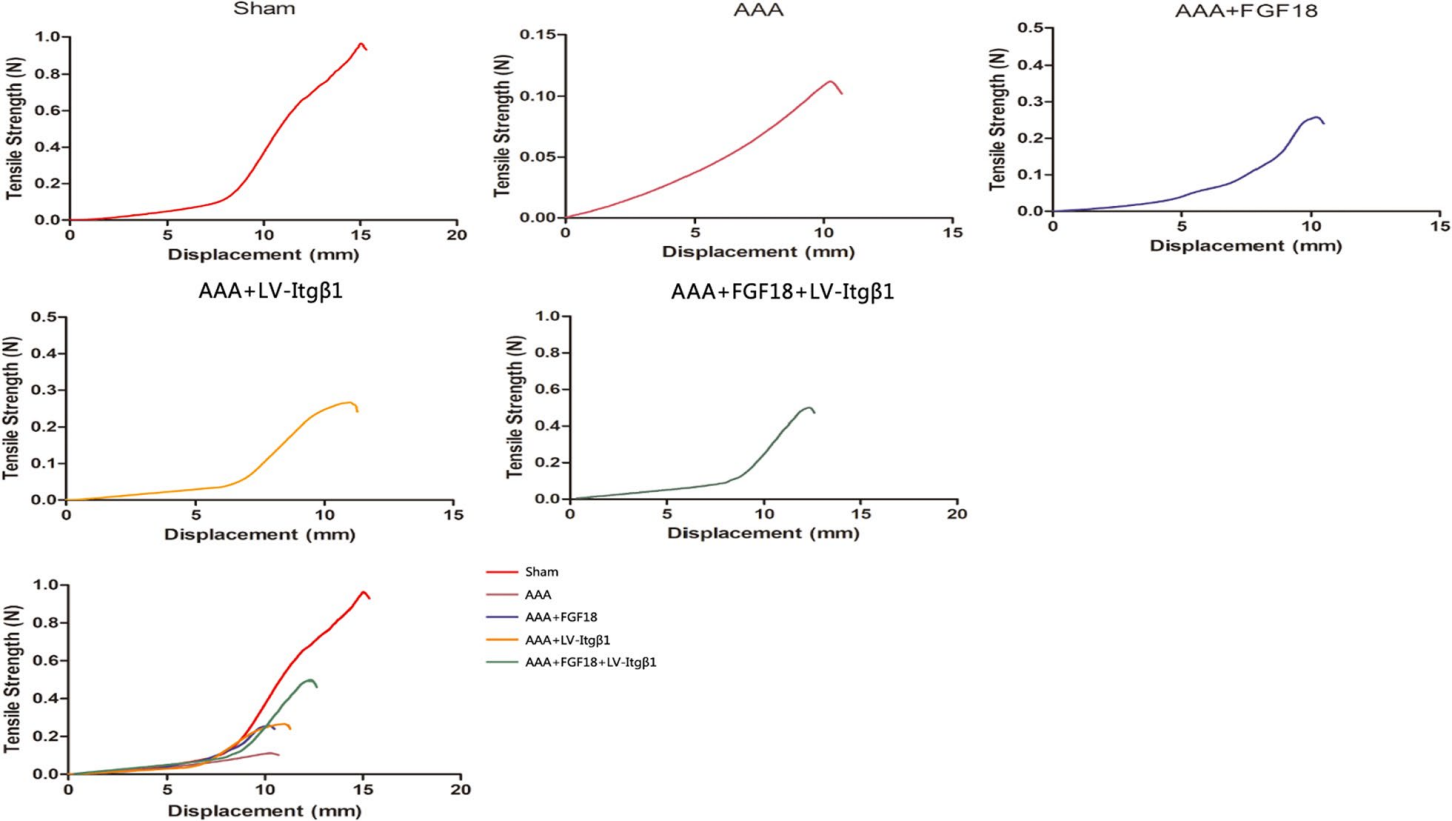
Tensile mechanical test of rat AAA tissues. Sham group: the aortic tensile resistance was the strongest, and the average maximum tensile force was 970 ± 26 mN; AAA group: the aortic tensile resistance was the lowest, and the average maximum tensile force was 111 ± 25 mN; the mean aortic maximum tensile forces of the AAA + LV-Itgβ1 and AAA + FGF18 groups were 254 ± 21 mN and 266 ± 23 mN, respectively; the aortic integrity to withstand stretch of the AAA + FGF18 + LV-Itgβ1 group was significantly higher than that of the AAA + LV-Itgβ1 and the AAA + FGF18 groups (*p* = 0.041, 0.032), and the average maximum stretching force was 493 ± 29 mN

The aortic integrity to withstand stretch in different groups was detected using the tensile mechanical test by testing how far the aorta can be stretched in length before breaking. The aortic integrity to withstand stretch of the AAA group was the lowest, whereas that of the Sham group was the highest. In addition, the aortic integrity to withstand stretch of the AAA + FGF18 + LV-Itgβ1 group was higher than that of the AAA + LV-Itgβ1 and AAA + FGF18 groups (*p* = 0.041, 0.032) (Fig. 6).

## Discussion

The development and popularity of imaging technologies, such as ultrasounds and CT scans, have increased the screening rate of aortic aneurysms in recent years. The existing guidelines only recommend surgical treatment for AAAs with a diameter greater than 5.5 cm and thoracic aortic aneurysms with a diameter greater than 6 cm. For aortic dilation diseases with a diameter less than 5 cm, only the control of high-risk factors and close follow-ups are recommended [22, 23]. The limitations resulting from follow-ups without intervention and the unpredictability of aortic aneurysm rupture call for continuous investigations on the biological repair methods that could delay or reverse the progress of aortic aneurysms [24].

The biological repair of aortic aneurysms has been proposed as a new direction in AAA treatment and denotes the use of biological interventions to promote extracellular matrix remodeling, inhibit apoptosis, promote cell proliferation, and delay the progression and reverse the pathophysiological process of aortic aneurysms [24]. Our study innovatively applied FGF18 + Itgβ1 to the AAA tissues of rats to explore the role of this regimen in AAA biological repair. The results showed that FGF18 promoted the expression of elastic fiber component proteins in ECs but could not do so in SMCs. However, FGF18 and Itgβ1 acted synergistically to promote the expression of elastic fiber component proteins in ECs and SMCs. Both Itgβ1 and FGF18 could further improve the proliferative and migratory abilities of ECs, but FGF18 alone could not promote the proliferative and migratory abilities of SMCs. FGF18 and Itgβ1 acted synergistically to promote the proliferative and migratory abilities of ECs and SMCs. FGF18 + Itgβ1 could also promote aortic tissue remodeling, improve SMC proliferation, delay the development of aortic dilation disease, and improve the aortic integrity to withstand stretch of AAA tissues. These results indicated that FGF18 + Itgβ1 could improve the biological repair efficacy of AAAs and delay their development.

Both ECs and SMCs are important components of the aortic wall. Therefore, many studies on the biological repair of aortic aneurysms have focused on the proliferation and functions of these cells. Elastic fibers are important cellular matrix components that are mainly secreted by ECs and SMCs and play a critical role in maintaining the stability of the aortic wall [25]. During the synthesis of elastic fibers, microfibrils are first synthesized by cells and attached to the cell surface to form a microfibril network for the elastic fibers. Subsequently, ELN aggregates on the cell surface and crosslinks with the microfibril network to form mature elastic fibers [26]. This implies that secretion and crosslinking with the cytoskeleton are equally important for the normal function of elastic fibers. FGF18 can promote the secretion of elastic proteins but not the connection between elastic fibers and the cytoskeleton [13]. Itgβ1 plays a key role in promoting the connection between the cytoskeleton and extracellular matrix [15, 16]. It not only mediates cell-to-cell and cell-to-extracellular matrix recognition and adhesion but also participates in bidirectional signal transduction by binding to corresponding ligands, thereby regulating cell proliferation, differentiation, and migration [27]. In this study, we observed that FGF18 and Itgβ1 work together to improve the expression of elastic proteins, promote their maturation and attachment to the cytoskeleton, and enhance the proliferative and migratory abilities of ECs and SMCs. All these processes work together to promote the biological repair of AAAs.

This study had a few limitations. We only explored the effects of FGF18 + Itgβ1 on the biological repair of AAAs at cellular and animal levels. As such, further research is necessary to assess whether this scheme can be applied in clinical practice. Additionally, this study only preliminarily explored the effect of FGF18 + Itgβ1 on the biological repair of AAAs. The biological repair of AAAs involves many intricate molecular mechanisms; therefore, further in-depth studies on the biological repair of AAAs are warranted.

## Conclusion

In this study, FGF18 + Itgβ1 was innovatively applied to the biological repair process of AAAs in rat models. The results revealed that FGF18 + Itgβ1 could improve the biological repair of AAAs by increasing the expression of elastic fiber component proteins, improving the migratory and proliferative abilities of ECs and SMCs, and improving aortic remodeling.

In addition, FGF18 + Itgβ1 may be potentially utilized for treating early detected small AAA in humans; however, further studies are needed to verify the therapeutic effects in animal species that are more easily compared to humans, such as pigs.

## Supplementary Information

**Additional file 1: Fig. S1**. Construction of the AAA model. (a) The abdominal aorta between the renal and iliac arteries was exposed and isolated from surrounding tissues using sterile rubber strips. (b, c) The abdominal aorta was wrapped in sterile cotton balls soaked in CaCl_2_ solution (0.8 mol/L). (d) Aneurysmal dilatation of the abdominal aorta. **Figure S2.** (a) Lentiviral transfection of ECs and SMCs: the fluorescence abundance of the LV-Con-RNAi group was higher than that of the LV-Itgβ1-RNAi group, whereas the fluorescence abundance of the LV-Itgβ1 group was higher than that of the LV-Con group. The fluorescence abundance of the NC + LV-Itgβ1 group was the highest, whereas that of the NC + LV-Itgβ1-RNAi group was the lowest. (b, c) WB and RT-qPCR assay for detecting Itgβ1 expression in ECs: Itgβ1 expression was lower in the NC + LV-Itgβ1-RNAi group than in the NC group; Itgβ1 expression was higher in the NC + LV-Itgβ1 group than in the NC group; Itgβ1 expression in the NC group was similar to that in the NC + LV-Con-RNAi and NC + LV-Con groups. (d, e) WB and RT-qPCR assay for detecting Itgβ1 expression in SMCs: Itgβ1 expression was lower in the NC + LV-Itgβ1-RNAi group than in the NC group; the Itgβ1 expression in the NC + LV-Itgβ1 group was higher than that in the NC group. **p < 0.01 versus NC group, ***p < 0.05 versus NC group. **Figure S3** Transwell chamber assay for detecting the migratory activity of ECs and SMCs. (a) Both FGF18 and Itgβ1 could enhance the migratory activity of ECs; there was no significant difference in the migratory activity of ECs between the NC and NC + LV-Con groups. Moreover, the migratory activity of ECs in the NC + LV-Itgβ1-RNAi group was the weakest, whereas it was strongest in the NC + LV-Itgβ1 + FGF18 group. (b) The migratory activity of SMCs was higher in the NC + LV-Itgβ1 group than in the NC and NC + LV-Con groups. There was no significant difference in the migratory activity of SMCs between the NC and NC + LV-Con groups. Moreover, the migratory ability of SMCs was higher in the NC + LV-Itgβ1 + FGF18 group than in the other groups; bar = 200 μm.

## Data Availability

All data generated or analyzed in this study are included in this published article.

## Abbreviations

AAA: Abdominal aortic aneurysm
SMC: Smooth muscle cell
EC: Endothelial cell
FGF18: Fibroblast growth factor 18
Itgβ1: Integrin β1
FBS: Fetal bovine serum
SDS-PAGE: Sodium dodecyl sulfate–polyacrylamide gel electrophoresis
RT-qPCR: Real-time quantitative reverse transcription-polymerase chain reaction
PBS: Phosphate-buffered saline
ELN: Elastin
FBN1: Fibrillin-1
FBLN5: Fibulin-5
GAPDH: Glyceraldehyde-3-phosphate dehydrogenase
AAA + LV-Itgβ1 group: AAA + lentivirus- Itgβ1 group
AAA + FGF18 + LV-Itgβ1 group: AAA + FGF18 + lentivirus-Itgβ1 group
