# Supplementary material for: The synergistic mechanism of fibroblast growth factor 18 and integrin β1 in rat abdominal aortic aneurysm repair
Source: BMC Cardiovasc Disord. 2022 Sep 17;22:415. doi: 10.1186/s12872-022-02851-y (PMC9482292; doi:10.1186/s12872-022-02851-y)
Supplement: Supplementary file 1 — Additional file 1: Fig. S1. Construction of the AAA model. (a) The abdominal aorta between the renal and iliac arteries was exposed and isolated from surrounding tissues using sterile rubber strips. (b, c) The abdominal aorta was wrapped in sterile cotton balls soaked in CaCl2 solution (0.8 mol/L). (d) Aneurysmal dilatation of the abdominal aorta. Figure S2. (a) Lentiviral transfection of ECs and SMCs: the fluorescence abundance of the LV-Con-RNAi group was higher than that of the LV-Itgβ1-RNAi group, whereas the fluorescence abundance of the LV-Itgβ1 group was higher than that of the LV-Con group. The fluorescence abundance of the NC + LV-Itgβ1 group was the highest, whereas that of the NC + LV-Itgβ1-RNAi group was the lowest. (b, c) WB and RT-qPCR assay for detecting Itgβ1 expression in ECs: Itgβ1 expression was lower in the NC + LV-Itgβ1-RNAi group than in the NC group; Itgβ1 expression was higher in the NC + LV-Itgβ1 group than in the NC group; Itgβ1 expression in the NC group was similar to that in the NC + LV-Con-RNAi and NC + LV-Con groups. (d, e) WB and RT-qPCR assay for detecting Itgβ1 expression in SMCs: Itgβ1 expression was lower in the NC + LV-Itgβ1-RNAi group than in the NC group; the Itgβ1 expression in the NC + LV-Itgβ1 group was higher than that in the NC group. **p < 0.01 versus NC group, ***p < 0.05 versus NC group. Figure S3 Transwell chamber assay for detecting the migratory activity of ECs and SMCs. (a) Both FGF18 and Itgβ1 could enhance the migratory activity of ECs; there was no significant difference in the migratory activity of ECs between the NC and NC + LV-Con groups. Moreover, the migratory activity of ECs in the NC + LV-Itgβ1-RNAi group was the weakest, whereas it was strongest in the NC + LV-Itgβ1 + FGF18 group. (b) The migratory activity of SMCs was higher in the NC + LV-Itgβ1 group than in the NC and NC + LV-Con groups. There was no significant difference in the migratory activity of SMCs between the NC and NC + LV-Con groups. Moreove [file 12872_2022_2851_MOESM1_ESM.pdf]

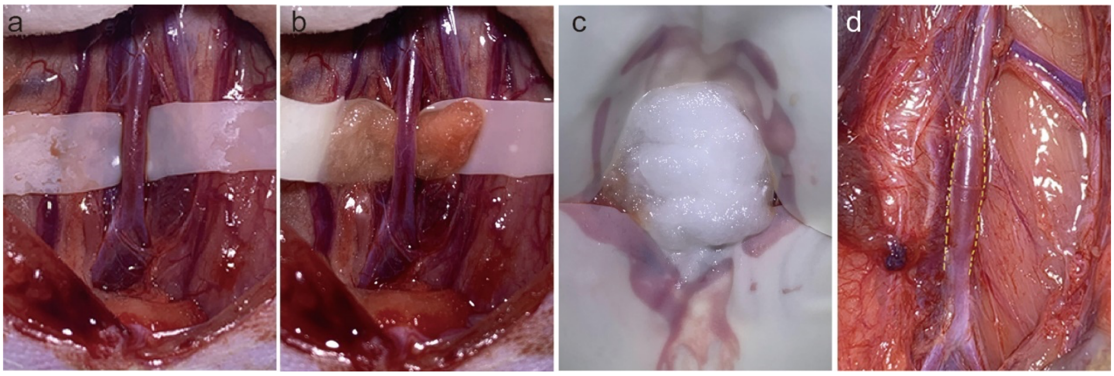

Supplementary Figure 1. Construction of the AAA model. (a) The abdominal aorta between the renal and iliac arteries was exposed and isolated from surrounding tissues using sterile rubber strips. (b, c) The abdominal aorta was wrapped in sterile cotton balls soaked in $CaCl_2$ solution (0.8 mol/L). (d) Aneurysmal dilatation of the abdominal aorta.

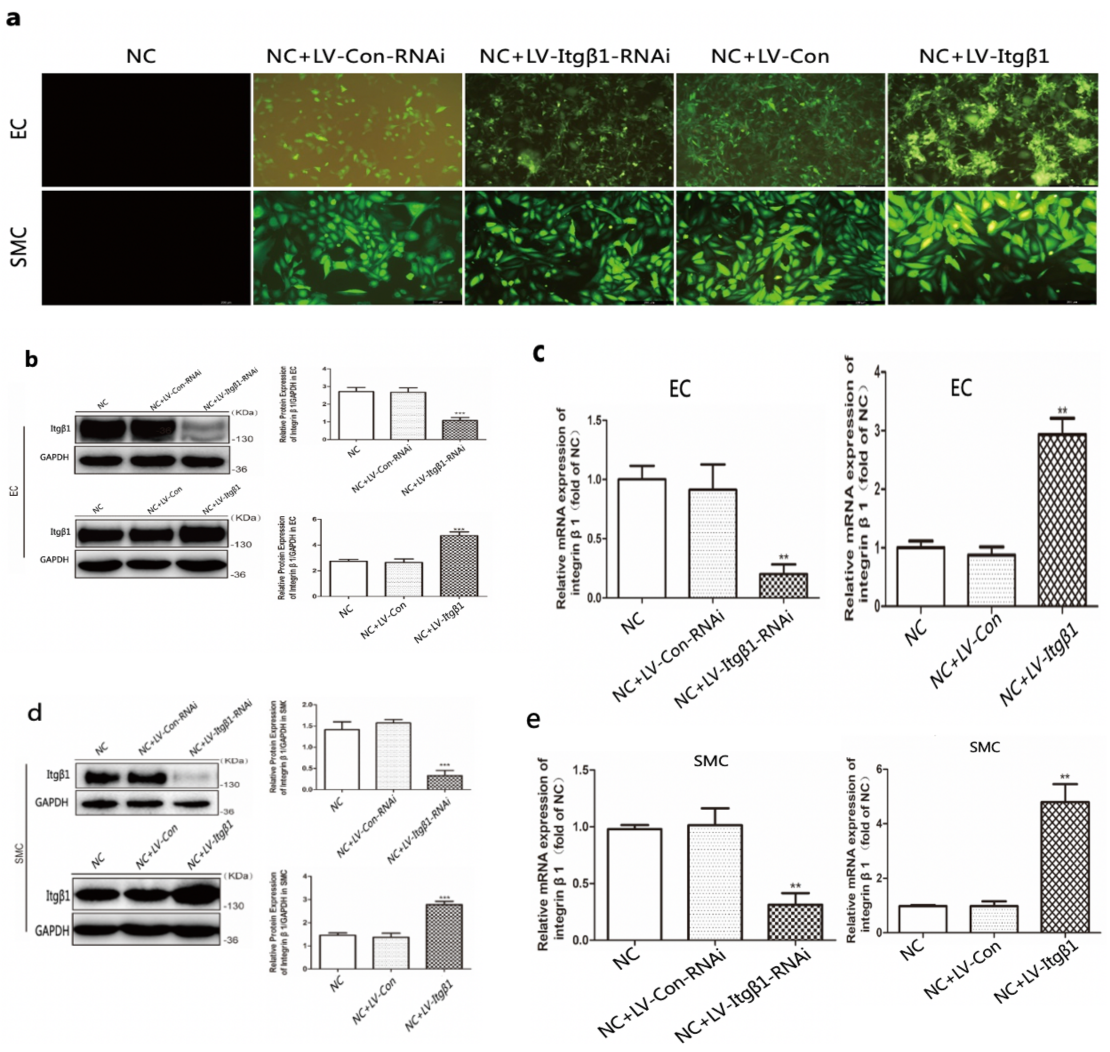

Supplementary Figure 2. (a) Lentiviral transfection of ECs and SMCs: the fluorescence abundance of the LV-Con-RNAi group was higher than that of the LV-Itgβ1-RNAi group, whereas the fluorescence abundance of the LV-Itgβ1 group was higher than that of the LV-Con group. The fluorescence abundance of the NC + LV-Itgβ1 group was the highest, whereas that of the NC + LV-Itgβ1-RNAi group was the lowest. (b, c) WB and RT-qPCR assay for detecting Itgβ1 expression in ECs: Itgβ1 expression was lower in the NC + LV-Itgβ1-RNAi group than in the NC group; Itgβ1 expression was higher in the NC + LV-Itgβ1 group than in the NC group; Itgβ1 expression in the NC group was similar to that in the NC + LV-Con-RNAi and NC + LV-Con groups. (d, e) WB and RT-qPCR assay for detecting Itgβ1 expression in SMCs: Itgβ1 expression was lower in the NC + LV-Itgβ1-RNAi group than in the NC group; the Itgβ1 expression in the NC + LV-Itgβ1 group was higher than that in the NC group. **$p < 0.01$ versus NC group, ***$p < 0.05$ versus NC group.

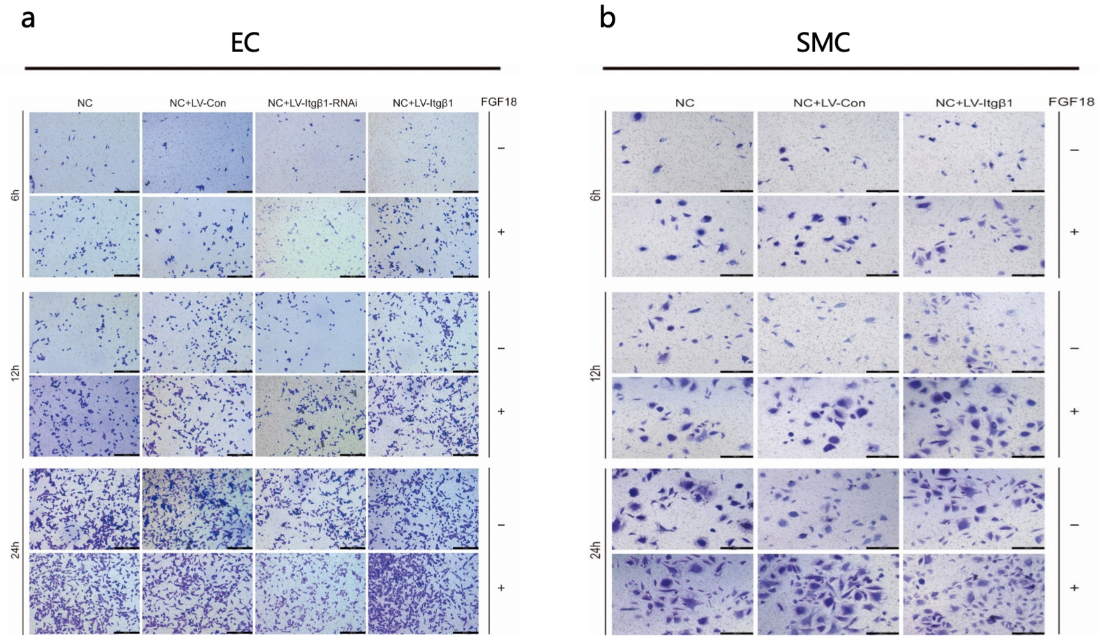

Supplementary Figure 3. Transwell chamber assay for detecting the migratory activity of ECs and SMCs. (a) Both FGF18 and Itgβ1 could enhance the migratory activity of ECs; there was no significant difference in the migratory activity of ECs between the NC and NC + LV-Con groups. Moreover, the migratory activity of ECs in the NC + LV-Itgβ1-RNAi group was the weakest, whereas it was strongest in the NC + LV-Itgβ1 + FGF18 group. (b) The migratory activity of SMCs was higher in the NC + LV-Itgβ1 group than in the NC and NC + LV-Con groups. There was no significant difference in the migratory activity of SMCs between the NC and NC + LV-Con groups. Moreover, the migratory ability of SMCs was higher in the NC + LV-Itgβ1 + FGF18 group than in the other groups; bar = 200 μm.
